# Responses in randomised groups of healthy, adult Labrador retrievers fed grain-free diets with high legume inclusion for 30 days display commonalities with dogs with suspected dilated cardiomyopathy

**DOI:** 10.1186/s12917-022-03264-x

**Published:** 2022-04-28

**Authors:** Anne Marie Bakke, Joshua Wood, Carina Salt, David Allaway, Matt Gilham, Gail Kuhlman, Tiffany Bierer, Richard Butterwick, Ciaran O’Flynn

**Affiliations:** 1 Waltham Petcare Science Institute, Mars Petcare UK, Freeby Lane, Waltham-on-the-Wolds, Melton Mowbray, Leicestershire LE14 4RT UK; 2 Mars Petcare US, Brentwood, TN USA

**Keywords:** Normocytic anemia, Hyperphosphatemia, Taurine status, Split peas, Lentils, Pulses, Flaxseed, DCM

## Abstract

Early responses in healthy adult dogs fed grain-free diets with high inclusion of split peas (20%) and lentils (40%) that may lead to canine diet-induced dilated cardiomyopathy (DCM) were investigated. To help understand the clinical relevance of the findings, a survey of electronic health records (EHR) was conducted of dogs with and without suspected DCM for comparison. Control and Test diets were fed to Labrador retriever dogs for 30 days (*n* = 5 and 6, respectively). Blood and urine samples collected at baseline and days 3, 14 and 28/30 were analyzed for hematology, clinical biochemistry and taurine concentrations. The EHRs of dogs at Banfield® Pet Hospitals in the 2-year period 2018-2019 were surveyed, revealing 420 dogs diagnosed with DCM, which were compared with 420 breed, gender and age-matched healthy control dogs. Compared to baseline values, feeding the Test diet for 28 days caused progressive, significant (*p* < 0.001) decreases in red blood cell counts (RBC), hematocrit and total hemoglobin by 7.7, 8.3 and 6.3%, respectively, and a 41.8% increase in plasma inorganic phosphate. Commonalities in these parameters were observed in clinical DCM cases. Regarding taurine status, Test dogs transiently increased whole-blood (23.4%) and plasma (47.7%) concentrations on day 14, while taurine:creatinine ratio in fresh urine and taurine in pooled urine were reduced by 77 and 78%, respectively, on day 28/30. Thus grain-free, legume-rich Test diets caused reduced RBC and hyperphosphatemia, findings also indicated in dogs with suspected DCM. Changes in taurine metabolism were indicated. The data will aid in generating hypotheses for future studies.

## Background

Recent years have shown an increasing trend in the use of less traditional (alternative) feed ingredients in pet foods. However, these may change the bioavailability or metabolism of nutrients and other dietary components, potentially leading to health issues. A possible current example is dilated cardiomyopathy (DCM) in dogs in the US following prolonged intake over months or years of grain-free, legume-rich diets [1, 2]. Historically, DCM was most prevalent in genetically predisposed large and giant breed dogs [3–5] or as a result of taurine deficiency [6, 7], which in dogs has been limited to certain breeds [7–15] suggesting genetic factors influencing taurine biosynthesis/metabolism. Although taurine deficiency was considered as a cause of the recent DCM cases, factors indicate that this may not always be the case: 1) reported diet, whole blood and plasma concentrations of taurine have in most cases been within recommended ranges [1, 2, 16, 17]; and 2) affected dogs are represented across a wider range of breeds/sizes and ages [1, 2, 18]. Further support for a dietary aetiology is provided by improvement of DCM when dogs are transitioned onto conventional, grain-inclusive diets, either with or without additional taurine supplementation [1, 2, 16, 19, 20]. However, the specific pathophysiological events leading to diet-associated DCM in dogs fed grain-free, legume-rich diets is currently not understood.

Although a number of clinical studies report implications of such diets on canine cardiac health [17–20], fewer report routine health parameters along with taurine status in dogs fed such diets [16, 21, 22].

To this end, a 30-day, longitudinal, small-scale study was initiated with the aim of understanding whether less conventional diets with high inclusion of legumes (60% total) lead to changes in routine hematology, clinical biochemistry, and whole blood, plasma and urinary taurine, the latter considered a more accurate indication of taurine status than blood concentrations [23]. The clinical relevance of the results of the responses were then examined by comparing them to those of dogs with DCM in their diagnosis, along with a breed, gender and age-matched set of healthy controls collected from a survey of electronic health records (EHRs) from Banfield® Pet Hospitals. The hypothesis tested was that routine health parameters and taurine status affected in healthy dogs fed diets with less conventional ingredients, including high levels of split peas and lentils, have clinical relevance for DCM development.

## Results

### Feeding study

#### Diets

The results of the nutrient analysis are provided in Table 1. Both Control and Test diets were nutritionally compliant as defined by guidance and regulatory agencies [24, 25], but they differed somewhat in specific nutrient levels. The Control diet was somewhat higher in carbohydrates and ash, while the Test diet was higher in protein, fat and contained twice as much crude fibre. Other nutrients of note were higher leucine, taurine, calcium (Ca), phosphorus (P) and vitamin A in the Control diet, whereas the Test diet contained higher concentrations of lysine, linoleic acid, alpha-linolenic acid, potassium, microminerals and most vitamins.

**Table 1:** Nutrient analysis results of the experimental diets as fed basis

Nutrients per 1000 kcal	Conventional Control diet	Grain-free Test diet
*Proximate composition*		
Moisture, %	7.9	9.8
NFE, g	136.41	130.14
Crude protein, g	65.34	69.42
Crude fat, g	27.45	30.70
Crude fibre, g	5.49	11.71
Ash, g	18.12	15.67
Calculated ME, kcal/kg	3643	3501
*Nutrient analysis*		
Arginine, g	3.60	4.77
Histidine, g	1.40	1.60
Isoleucine, g	2.31	2.68
Leucine, g	5.93	4.60
Lysine, g	3.02	4.34
Methionine, g	1.25	1.43
Methionine + cystine, g	2.04	2.06
Phenylalanine, g	2.83	3.00
Phenylalanine+tyrosine, g	4.80	4.80
Taurine, g	0.14	0.03
Threonine, g	2.31	2.46
Tryptophan, g	0.60	0.69
Valine, g	2.96	3.00
Linoleic+Arachidonic acid, g	7.58	11.85
Linoleic acid, g	7.44	11.80
Arachidonic acid, g	0.14	0.06
Alpha linolenic acid, g	0.38	4.66
EPA + DHA, g	0.11	0.11
Calcium, g	3.57	1.81
Phosphorus, g	2.50	1.39
Ca: P ratio	1.43	1.30
Potassium, g	1.81	3.21
Sodium, g	1.27	0.82
Chloride, g	2.39	2.43
Magnesium, g	0.32	0.37
Copper, mg	4.57	6.26
Iodine, mg	0.19	0.60
Iron, mg	26.6	56.3
Manganese, mg	3.52	9.03
Selenium, μg	175.7	322.8
Zinc, mg	65.9	101.4
Vitamin A, IU	1641.7	4284.5
Vitamin D, IU	257.2	464.0
Vitamin E, IU	51.06	35.13
Thiamine, mg	2.05	4.11
Riboflavin, mg	12.08	18.02
Pantothenic acid, mg	13.31	31.42
Pyridoxine, mg	10.84	17.77
Vitamin B12, μg	15.54	31.71
Niacin, mg	81.26	107.40
Folic acid, μg	204.5	262.8
Biotin, μg	576.5	991.1
Choline, mg	475.8	1028.6
Vitamin K, mg	–	0.29

Besides the differences in the animal-derived ingredients and legume content, a relatively high level of flaxseed meal was also included in the Test diet. Analysis for cyanogenic glycosides present in flaxseed revealed differences between diets: the Control diet contained 13 mg/kg linamarin, while linustatin and neolinustatin concentrations were below detection limit (< 10 mg/kg). The Test diet contained < 10 mg/kg linamarin, 103 mg/kg linustatin and 128 mg/kg neolinustatin.

Both diets were palatable and well-accepted by the dogs during the trial, with no reports of meal refusals or apparent adverse effects on the dogs’ general health and well-being.

#### Blood parameters and taurine status

Table 2 summarizes the hematology data. Red blood cell counts (RBC) were significantly (*p* < 0.05) reduced in Test dogs compared to baseline and dogs fed the Control diet on days 3, 14 and 28, with five and six of six Test dogs demonstrating counts below the lower limit of the reference range (6∙10^12^/L) on days 14 and 28, respectively. Concomitant reductions in hematocrit (HCT) (*p* < 0.05) were observed, while the mean cell volume (MCV) and reticulocyte distribution width (RDW) (Table 2) were not or only transiently affected. Blood total hemoglobin (HGB) concentration also decreased significantly from baseline on day 28 in the dogs fed the Test diet but remained within the reference range. Mean cell hemoglobin (MCH) and mean cell hemoglobin concentration (MCHC) were generally above the upper limit of the respective reference ranges in both Control and Test-fed dogs. And MCHC increased significantly from baseline on all sampling days reaching an apparent plateau from day 15 for Test dogs. Total white blood cell counts (WBC) were below or within respective physiological ranges, despite some transient, statistically significant diet differences on day 3 (Table 2). Mean platelet volume (MPV) increased compared to baseline in the Test group over the course the feeding trial, significantly so on days 14 and 28, but also remained within the reference range.

**Table 2:** Mean fasted hematology results with 95% confidence intervals for each diet group (Control, n = 5; Test, *n* = 6) and sampling time (baseline and 3, 14 and 28 days), as well as results of the statistical analysis (*p*-values). In the left column, units for each parameter are provided in parentheses and internal lab reference ranges for each parameter are provided in italics

Parameter	Sampling time	Control		Test		Statistical analysis	
		Mean	95% CI	Mean	95% CI	Diff. within Test diet	Diff. between diets^†^
Red blood cell count (RBC) (10^12^/L) *6-9*	Baseline	5.86	(5.60, 6.13)	5.82	(5.58, 6.06)	–	–
	Day 3	6.09*	(5.82, 6.37)	**5.74**	(5.50, 5.98)	NS	0.034
	Day 14	**5.97**	(5.71, 6.24)	5.63*	(5.40, 5.86)	0.049	0.036
	Day 28	**5.86**	(5.60, 6.13)	5.37*	(5.15, 5.59)	< 0.001	< 0.001
Hematocrit (HCT) (%) *40-55*	Baseline	45.1	(43.1, 47.1)	44.8	(43.0, 46.6)	–	–
	Day 3	45.7	(43.7, 47.7)	43.4*	(41.6, 45.1)	0.035	NS
	Day 14	**45.3**	(43.4, 47.3)	42.6*	(40.9, 44.3)	< 0.001	0.011
	Day 28	**45.3**	(43.3, 47.3)	41.1*	(39.5, 42.8)	< 0.001	< 0.001
Total hemoglobin (HGB) (g/dL) *12-19*	Baseline	15.9	(15.2, 16.7)	15.8	(15.1, 16.4)	–	–
	Day 3	16.6*	(15.8, 17.3)	15.8	(15.1, 16.5)	NS	NS
	Day 14	16.2	(15.5, 17.0)	15.3	(14.7, 15.9)	NS	NS
	Day 28	**16.4**	(15.7, 17.2)	14.8*	(14.2, 15.4)	< 0.001	< 0.001
Mean cell volume (MCV) (fL) *60-77*	Baseline	76.9	(75.4, 78.4)	77.0	(75.6, 78.4)	–	–
	Day 3	75.1*	(73.6, 76.6)	75.6*	(74.2, 77.0)	< 0.001	NS
	Day 14	75.9*	(74.4, 77.4)	75.7*	(74.3, 77.1)	< 0.001	NS
	Day 28	77.3	(75.8, 78.8)	76.6	(75.2, 78.0)	NS	NS
Mean cell hemoglobin (MCH) (pg) *17-23*	Baseline	27.2	(26.5, 27.9)	27.1	(26.5, 27.7)	–	–
	Day 3	27.2	(26.6, 27.9)	27.5	(26.9, 28.2)	NS	NS
	Day 14	27.2	(26.5, 27.8)	27.2	(26.6, 27.8)	NS	NS
	Day 28	28.0*	(27.3, 28.7)	27.5	(26.9, 28.1)	NS	NS
Mean cell hemoglobin concentra-tion (MCHC) (g/dL) *31-34*	Baseline	35.4	(34.7, 36.0)	35.2	(34.6, 35.8)	–	–
	Day 3	36.2*	(35.6, 36.9)	36.4*	(35.9, 37.0)	< 0.001	NS
	Day 14	35.8	(35.2, 36.4)	35.9*	(35.4, 36.5)	0.023	NS
	Day 28	36.2*	(35.6, 36.8)	35.9*	(35.4, 36.5)	0.021	NS
Red cell distribution width (RDW) (%) *14-17*	Baseline	13.3	(12.7, 13.9)	13.7	(13.1, 14.3)	–	–
	Day 3	14.0*	(13.3, 14.6)	**13.6**	(13.0, 14.2)	NS	0.003
	Day 14	14.4*	(13.8, 15.1)	**13.8**	(13.2, 14.3)	NS	< 0.001
	Day 28	14.2*	(13.6, 14.9)	**13.6**	(13.0, 14.2)	NS	< 0.001
White blood cell count (WBC) (10^9^/L) *6-12*	Baseline	5.40	(4.25, 6.85)	5.41	(4.35, 6.72)	–	–
	Day 3	6.28*	(4.95, 7.97)	**5.35**	(4.30, 6.65)	NS	0.049
	Day 14	5.85	(4.61, 7.43)	5.79	(4.66, 7.20)	NS	NS
	Day 28	6.01	(4.73, 7.63)	5.53	(4.45, 6.87)	NS	NS
Lymphocyte count (10^9^/L) *1.2-3.2*	Baseline	1.44	(1.17, 1.71)	1.36	(1.17, 1.71)	–	–
	Day 3	1.60	(1.33, 1.87)	1.46	(1.17, 1.71)	NS	NS
	Day 14	1.41	(1.14, 1.68)	1.43	(1.17, 1.71)	NS	NS
	Day 28	1.66	(1.39, 1.93)	1.42	(1.17, 1.71)	NS	NS
Monocyte count (10^9^/L) *0.3-0.8*	Baseline	0.55	(0.43, 0.67)	0.53	(0.42, 0.64)	–	–
	Day 3	0.61	(0.49, 0.73)	0.60*	(0.49, 0.71)	0.044	NS
	Day 14	0.60	(0.48, 0.72)	0.57	(0.46, 0.68)	NS	NS
	Day 28	0.64*	(0.52, 0.76)	0.58	(0.47, 0.69)	NS	NS
Eosinophil count (10^9^/L) 1.2-6.8	Baseline	3.46	(2.51, 4.78)	3.50	(2.61, 4.70)	–	–
	Day 3	3.98	(2.89, 5.50)	3.28	(2.45, 4.41)	NS	NS
	Day 14	3.80	(2.75, 5.24)	3.79	(2.83, 5.09)	NS	NS
	Day 28	3.62	(2.62, 5.00)	3.52	(2.63, 4.73)	NS	NS
Platelet count (PLT) (10^9^/L) *150-500*	Baseline	218	(180, 255)	186	(151, 220)	–	–
	Day 3	201	(164, 239)	188	(154, 222)	NS	NS
	Day 14	211	(174, 249)	173	(139, 208)	NS	NS
	Day 28	199	(162, 237)	159	(124, 193)	NS	NS
Mean platelet volume (MPV) (mm^3^) *6.7-11.1*	Baseline	7.35	(6.54, 8.16)	7.77	(7.03, 8.50)	–	–
	Day 3	7.30	(6.49, 8.11)	8.07	(7.33, 8.80)	NS	NS
	Day 14	**7.34**	(6.53, 8.15)	8.52*	(7.79, 9.26)	< 0.001	< 0.001
	Day 28	**7.31**	(6.50, 8.12)	8.63*	(7.90, 9.37)	< 0.001	< 0.001

*NS* no significant difference (*p* > 0.05)

^*^indicate a significant change (*p* < 0.05) from baseline within diet group

^†^indicate significant differences (*p* < 0.05) in the change from baseline between diet groups, also indicated with bold values

Clinical biochemistry data are summarized in Table 3. Plasma inorganic P concentrations were significantly increased in dogs fed the Test diet compared to baseline, and significantly more than Control-fed dogs, at all three sampling times after baseline. Half the dogs exhibited P concentrations at or above the upper limit of the reference range at days 14 and 28. Plasma total Ca and whole blood ionized Ca (iCa) were transiently and significantly decreased in dogs fed the Test diet on days 3 and 14, with half the dogs exhibiting Ca concentrations below the lower limit of the reference range at those time points. On day 28, however, Ca and iCa concentrations had recovered and did not differ from baseline values (*p* > 0.05). Total protein, albumin and globulin were not affected by the Test diet. Cholesterol decreased from baseline in the dogs fed the Test diet. However, concentrations were within the reference range and significant differences between diets were not detected (Table 3). Liver enzymes alanine aminotransferase (ALT) and aspartate aminotransferase (AST) activities were increased in all dogs, significantly for AST on all sampling days for the Control group dogs and at day 14 in the Test dogs. AST activities were also often above the upper limit of the reference range in dogs in both diet groups, with all Test group dogs exhibiting values above 41 U/L at day 14. However, no differences between diets were observed and the activities were apparently decreasing in most dogs in both diet groups on day 28.

**Table 3:** Mean fasted clinical biochemistry results with 95% confidence intervals for each diet group (Control, *n* = 5; Test, *n* = 6) and sampling time (baseline and 3, 14 and 28 days), as well as results of the statistical analysis (*p*-values). In the left column, units for each parameter are provided in parentheses and IDEXX lab reference ranges (unless otherwise specified) for each parameter are provided in italics

Parameter	Sampling time	Control		Test		Statistical analysis	
		Mean	95% CI	Mean	95% CI	Diff. within Test diet	Diff. between diets^†^
Total protein (g/L) *54.9-75.3*	Baseline	56.1	(54.0, 58.2)	57.4	(55.5, 59.4)	–	–
	Day 3	55.9	(53.8, 58.1)	56.2	(54.3, 58.2)	NS	NS
	Day 14	56.6	(54.5, 58.8)	56.3	(54.4, 58.3)	NS	NS
	Day 28	57.5	(55.4, 59.6)	56.8	(54.8, 58.7)	NS	NS
Albumin (g/L) *26.3-38.2*	Baseline	29.7	(27.9, 31.5)	29.7	(28.0, 31.3)	–	–
	Day 3	29.7	(27.9, 31.5)	29.3	(27.7, 31.0)	NS	NS
	Day 14	30.2	(28.4, 32.0)	29.4	(27.8, 31.1)	NS	NS
	Day 28	30.9*	(29.1, 32.7)	29.9	(28.2, 31.5)	NS	NS
Globulin (g/L) *23.4-42.2*	Baseline	26.4	(24.8, 28.1)	27.7	(26.2, 29.3)	–	–
	Day 3	26.2	(24.5, 27.9)	26.9	(25.3, 28.4)	NS	NS
	Day 14	26.4	(24.8, 28.1)	26.9	(25.4, 28.4)	NS	NS
	Day 28	26.6	(24.9, 28.3)	26.9	(25.3, 28.4)	NS	NS
Alb:glob ratio *0.7-1.4*	Baseline	1.13	(1.02, 1.23)	1.07	(0.98, 1.17)	–	–
	Day 3	1.14	(1.03, 1.25)	1.10	(1.00, 1.20)	NS	NS
	Day 14	1.15	(1.04, 1.25)	1.10	(1.00, 1.20)	NS	NS
	Day 28	1.17	(1.06, 1.27)	1.12	(1.02, 1.22)	NS	NS
Alanine aminotransferase (ALT) (U/L) *19.8-124*	Baseline	36.6	(26.4, 50.6)	44.5	(33.1, 59.8)	–	–
	Day 3	45.1	(32.6, 62.5)	59.9	(44.5, 80.6)	NS	NS
	Day 14	47.7	(34.5, 66.0)	52.0	(38.7, 70.0)	NS	NS
	Day 28	49.9	(36.1, 69.0)	48.9	(36.3, 65.7)	NS	NS
Aspartate aminotransferase (AST) (U/L) *14-41*	Baseline	31.9	(24.7, 41.3)	39.1	(30.9, 49.4)	–	–
	Day 3	39.2*	(30.4, 50.7)	43.0	(34.1, 54.4)	NS	NS
	Day 14	41.2*	(31.8, 53.2)	50.1*	(39.6, 63.3)	< 0.001	NS
	Day 28	37.3*	(28.9, 48.2)	44.2	(35.0, 55.9)	NS	NS
Alkaline phosphatase (ALP) (U/L) *< 130*	Baseline	41.0	(28.9, 53.1)	40.7	(29.6, 51.7)	–	–
	Day 3	48.2	(36.1, 60.3)	53.8*	(42.8, 64.9)	0.002	NS
	Day 14	46.4	(34.3, 58.5)	58.2*	(47.1, 69.2)	< 0.001	NS
	Day 28	44.8	(32.7, 56.9)	52.8*	(41.8, 63.9)	0.004	NS
Bone-specific ALP (U/L)	Baseline	12.6	(10.1, 15.2)	12.3	(10.0, 14.6)	**–**	**–**
	Day 3	13.1	(10.6, 15.7)	12.9	(10.6, 15.2)	NS	NS
	Day 14	13.2	(10.7, 15.7)	12.3	(10.0, 14.6)	NS	NS
	Day 28	13.2	(10.7, 15.7)	11.8	(9.5, 14.1)	NS	NS
Creatinine (μmol/L) *44-133*	Baseline	90.7	(79.3, 104)	87.8	(77.7, 99.2)	–	–
	Day 3	85.9	(75.1, 98.3)	77.7*	(68.7, 87.8)	< 0.001	NS
	Day 14	91.6	(80.1, 105.0)	82.8	(73.3, 93.6)	NS	NS
	Day 28	94.1	(82.3, 108.0)	87.1	(77.1, 98.5)	NS	NS
Urea (mmol/L) *3.1-10.1*	Baseline	5.50	(4.41, 6.59)	5.56	(4.56, 6.56)	–	–
	Day 3	5.14	(4.05, 6.24)	4.76	(3.77, 5.76)	NS	NS
	Day 14	5.64	(4.55, 6.74)	4.71	(3.71, 5.70)	NS	NS
	Day 28	5.61	(4.51, 6.70)	5.09	(4.09, 6.08)	NS	NS
Cholesterol (mmol/L) *3.20-6.20*	Baseline	4.25	(3.46, 5.22)	4.68	(3.88, 5.64)	–	–
	Day 3	4.11	(3.35, 5.04)	4.21*	(3.49, 5.08)	0.005	NS
	Day 14	4.05	(3.30, 4.98)	4.02*	(3.33, 4.84)	< 0.001	NS
	Day 28	4.15	(3.38, 5.10)	4.14*	(3.43, 5.00)	< 0.001	NS
Triglycerides (mmol/L) *0.30-1.20*	Baseline	0.61	(0.48, 0.77)	0.49	(0.39, 0.61)	–	–
	Day 3	0.59	(0.46, 0.75)	0.47	(0.38, 0.59)	NS	NS
	Day 14	0.65	(0.51, 0.83)	0.51	(0.41, 0.64)	NS	NS
	Day 28	0.68	(0.54, 0.86)	0.51	(0.41, 0.63)	NS	NS
Glucose (mmol/L) *3.6-7.0*	Baseline	5.60	(5.12, 6.09)	5.34	(4.89, 5.78)	–	–
	Day 3	5.50	(5.01, 5.98)	5.19	(4.75, 5.63)	NS	NS
	Day 14	5.42	(4.94, 5.90)	4.93	(4.49, 5.37)	NS	NS
	Day 28	5.65	(5.17, 6.14)	4.82*	(4.38, 5.26)	0.017	NS
Calcium (mmol/L) *2.36-2.84*	Baseline	2.48	(2.40, 2.56)	2.43	(2.36, 2.50)	–	–
	Day 3	2.45	(2.37, 2.53)	2.38	(2.30, 2.45)	NS	NS
	Day 14	2.41	(2.33, 2.49)	2.36*	(2.28, 2.43)	0.043	NS
	Day 30	2.50	(2.42, 2.58)	2.43	(2.36, 2.51)	NS	NS
Ionised calcium (mmol/L) *1.19-1.33*	Baseline	1.32	(1.29, 1.35)	1.31	(1.28, 1.33)	–	–
	Day 3	1.27*	(1.24, 1.30)	1.26*	(1.23, 1.28)	< 0.001	NS
	Day 14	1.32	(1.29, 1.35)	1.27*	(1.24, 1.30)	0.004	NS
	Day 30	1.32	(1.29, 1.35)	1.30	(1.27, 1.33)	NS	NS
Inorganic phosphate (P) (mmol/L) *0.80-1.60*	Baseline	1.26	(1.10, 1.43)	1.22	(1.08, 1.37)	–	–
	Day 3	**1.18**	(1.04, 1.35)	**1.56**	(1.38, 1.76)	< 0.001	< 0.001
	Day 14	**1.16**	(1.02, 1.33)	**1.65**	(1.46, 1.85)	< 0.001	< 0.001
	Day 30	**1.25**	(1.10, 1.43)	**1.73**	(1.54, 1.95)	< 0.001	< 0.001
N-terminal pro b-type natriuretic peptide (NTproBNP) (pmol/L) *275-2100* [26]	Baseline	738	(335, 1639)	521	(236, 1150)	–	–
	Day 28	549	(249, 1210)	640	(275, 1490)	NS	NS

*NS* no significant difference (*p* > 0.05)

^*^indicate a significant change (*p* < 0.05) from baseline within diet group

^†^indicate significant differences (*p* < 0.05) in the change from baseline between diet groups, also indicated with bold values

Alkaline phosphatase (ALP) activities were also increased in the Test group dogs from baseline at all sampling times, although within the reference range for all dogs. Bone-specific ALP activities did not reveal differences from baseline or between groups. Creatinine concentrations were transiently decreased in Test dogs on day 3, while no significant changes were observed in urea, and all values for all dogs were within the respective reference ranges (Table 3). Signs of gross hemolysis in the plasma samples were not observed during analysis.

The heart specific biomarker N-terminal pro b-type natriuretic peptide (NTproBNP) concentration in serum, which indicates myocardial stretch when elevated, was not significantly affected by diet and remained within the reference range suggested for Labrador retrievers of 275-2100 pmol/L [26] for all dogs. Nor did any of the dogs fed the control (*n* = 2) or Test diet (*n* = 4) show changes of concern in cardiac parameters as evaluated by the radiographer conducting the ultrasound scans (data not shown).

Table 4 summarizes taurine status. Compared to baseline values, whole blood taurine concentrations increased significantly in both Control and Test group dogs, significantly on days 14 and 28 for Control group, while in Test group dogs, a transient increase was observed on day 14 only. No significant differences between diet groups were observed for whole blood, while plasma concentrations increased more in Test compared to Control on day 14. Taurine in fresh urine varied widely, and a significant decrease from baseline was only detected in Test dogs on day 3. When related to creatinine concentrations (taurine:creatinine ratios; Table 4), however, a significant, decrease was detected in Test

**Table 4:** Taurine status (in μmol/L) as assessed in whole blood, plasma and urine (fresh and pooled). Values are means with 95% confidence intervals for each diet group (Control, *n* = 5; Test, *n* = 6) and sampling time (baseline and 3, 14 and 28/30 days), as well as results of the statistical analysis (es). Parameters measured in fresh urine samples are from fasted dogs

Parameter	Sampling time	Control		Test		Statistical analysis	
		Mean	95% CI	Mean	95% CI	Diff. within Test diet	Diff. between diets^†^
Whole blood taurine	Baseline	181	(155, 211)	205	(178, 236)	–	–
	Day 3	188	(161, 219)	205	(179, 236)	NS	NS
	Day 14	233*	(200, 271)	253*	(220, 291)	< 0.001	NS
	Day 28	217*	(186, 253)	211	(183, 242)	NS	NS
Plasma taurine	Baseline	98.2	(65.6, 147)	109	(75.4, 157)	–	–
	Day 3	92.1	(61.5, 138)	120	(83.0, 173)	NS	NS
	Day 14	**88.7**	(59.2, 133)	161*	(111, 233)	0.011	0.048
	Day 28	126	(84.3, 189)	120	(83.0, 173)	NS	NS
Fresh urine taurine	Baseline	523	(152, 1800)	740	(239, 2290)	–	–
	Day 3	**1350**	(391, 4640)	**326**	(105, 1010)	NS	0.033
	Day 14	1250	(363, 4310)	1540	(499, 4770)	NS	NS
	Day 28	547	(159, 1890)	451	(146, 1400)	NS	NS
Fresh urine Tau: Creat ratio	Baseline	0.112	(0.039, 0.321)	0.114	(0.044, 0.298)	–	–
	Day 3	**0.164**	(0.057, 0.469)	0.028*	(0.011, 0.074)	< 0.001	< 0.001
	Day 14	0.131	(0.046, 0.374)	0.106	(0.041, 0.277)	NS	NS
	Day 28	0.053	(0.019, 0.153)	0.026*	(0.010, 0.067)	< 0.001	NS
Pooled urine taurine	Baseline	598	(214, 1670)	1300	(508, 3330)	–	–
	Day 30	**611**	(218, 1710)	282*	(110, 721)	< 0.001	< 0.001

*NS* no significant difference (*p* > 0.05)

^*^indicate a significant change (*p* < 0.05) from baseline within diet group

^†^indicate significant differences (*p* < 0.05) in the change from baseline between diet groups, also indicated with bold values

group dogs on both day 3 and 28 compared to baseline, and a diet group difference was observed on day 3. This was substantiated by similar reductions in taurine concentrations measured in pooled urine at baseline and day 30. In the dogs fed the Test diet, pooled urine taurine concentration decreased to 21% of baseline, while the mean taurine-to-creatinine ratio value measured on day 28 decreased to 23%.

### EHR interrogation

**Fig. 1:**
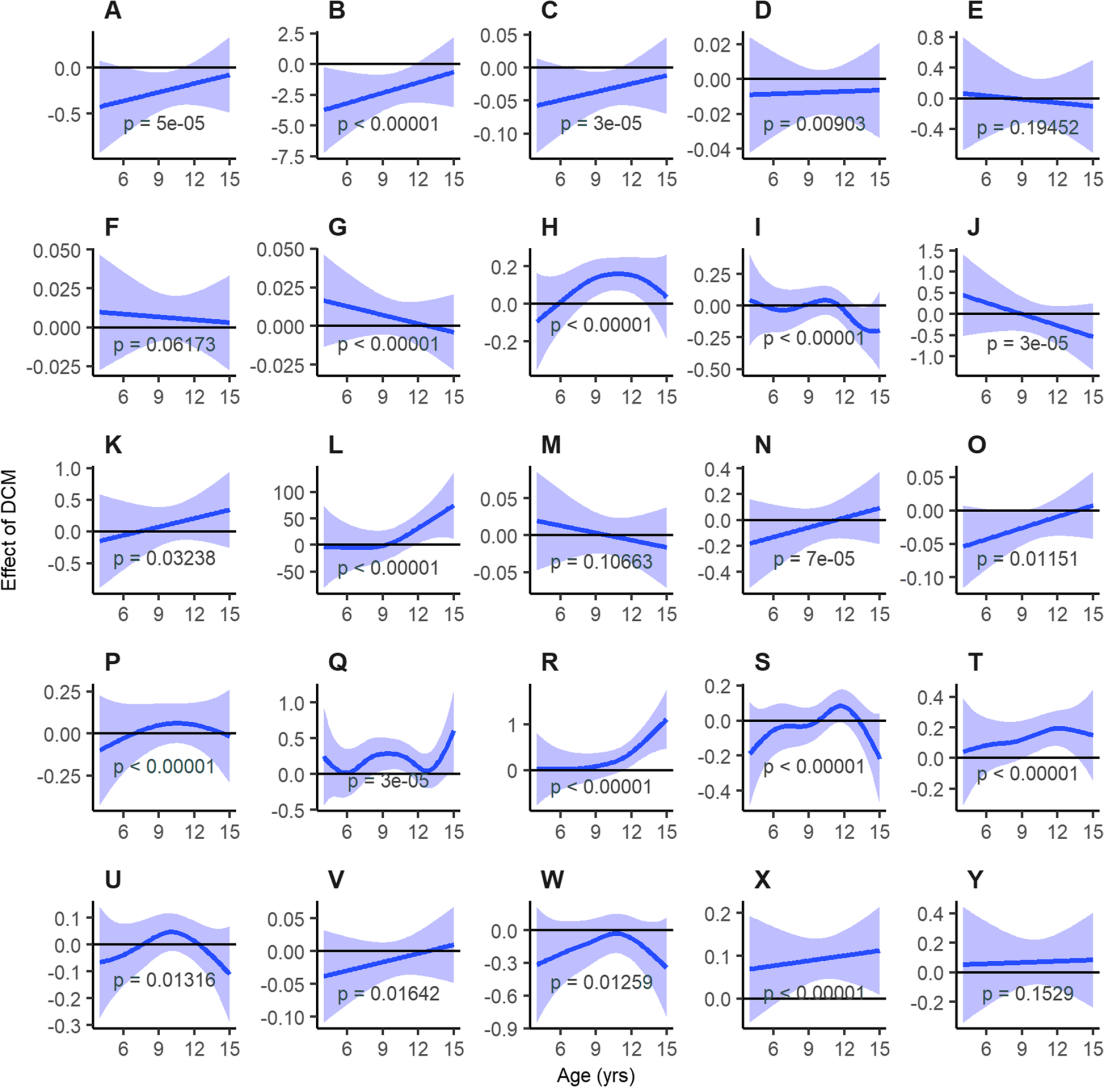
Estimated relative effect of DCM by blood analyte and age with confidence intervals (shaded areas). The relative difference between dogs with DCM (blue lines) and age and breed-matched healthy control dogs (black lines; set at 0) are indicated for each analyte and by age. The significance level was set at *p* < 0.002 (see text for explanation). Panes are as follows: **A** - Red Blood Cell Count, **B** - Hematocrit, **C** - Log(Total Hemoglobin), **D** - Log(Mean Cell Volume), **E** – Mean Cell Hemoglobin, **F** - Log(Mean Cell Hemoglobin Concentration), **G** - Log(Reticulocyte Distribution Width), **H** - Log(White Blood Cell Count), **I** - Log(Lymphocyte %), **J** - Monocyte %, **K** - Eosinophil %, **L** – Platelet Count, **M** - Log(Mean Platelet Volume), **N** - Total Protein, **O** - Log(Albumin), **P** - Globulin, **Q** - Log(Alanine Aminotransferase), **R** - Log(Alkaline Phosphatase), **S** - Log(Creatinine), **T** - Log(Blood Urea Nitrogen), **U** - Log(Cholesterol), **V** - Log(Glucose), **W** - Calcium, **X** - Log(Inorganic Phosphate), **Y** - Log(Total Bilirubin)

**Fig. 2:**
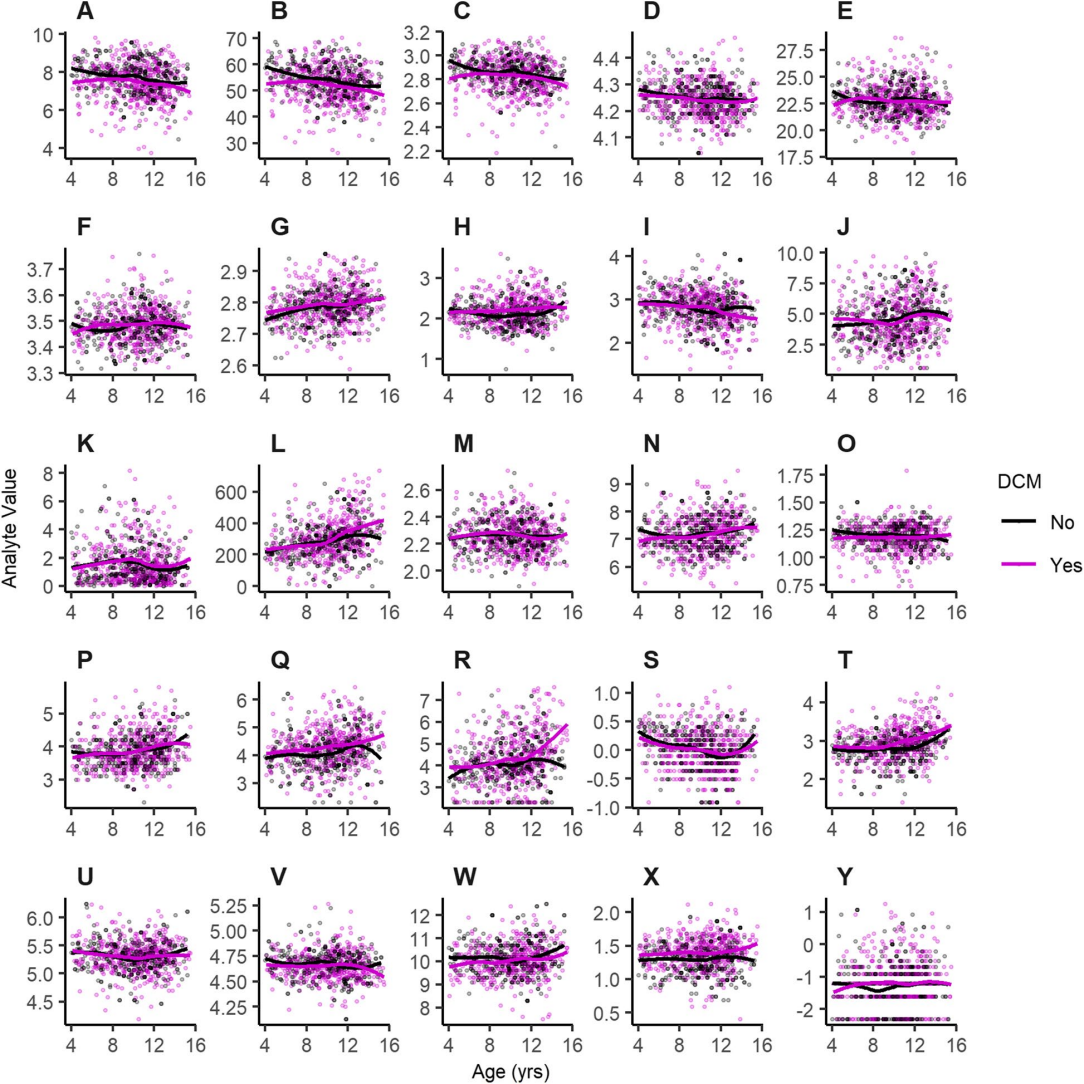
Scatter plots of data for each analyte by age for dogs with DCM (purple) and healthy controls (black). Corresponding loess smoothed trend lines are also provided. Panes are as follows: **A** – Red Blood Cell Count, **B** - Hematocrit, **C** - Log(Total Hemoglobin), **D** - Log(Mean Cell Volume), **E** – Mean Cell Hemoglobin, **F** - Log(Mean Cell Hemoglobin Concentration), **G** - Log(Reticulocyte Distribution Width), **H** - Log(White Blood Cell Count), **I** - Log(Lymphocyte %), **J** - Monocyte %, **K** - Eosinophil %, **L** – Platelet Count, **M** - Log(Mean Platelet Volume), **N** - Total Protein, **O** - Log(Albumin), **P** - Globulin, **Q** - Log(Alanine Aminotransferase), **R** - Log(Alkaline Phosphatase), **S** - Log(Creatinine), **T** - Log(Blood Urea Nitrogen), **U** - Log(Cholesterol), **V** - Log(Glucose), **W** - Calcium, **X** - Log(Inorganic Phosphate), **Y** - Log(Total Bilirubin)

Figure 1 shows the estimated relative differences and Fig. 2 shows the raw scatter plots for dogs with DCM compared to age and breed-matched controls without DCM for hematology and clinical biochemistry parameters by analyte and age. The HCT, lymphocyte, and albumin data did not converge when the model was fitted. Therefore, the model and subsequent interpretation is unreliable for these analytes.

Significant relative differences (*p* < 0.002) between dogs with DCM and healthy controls for the hematology parameters RBC, HCT, HGB, RDW, WBC, lymphocytes, monocytes, and platelet counts were observed. Compared to matched controls, RBC count, HCT and HGB were reduced, especially in younger dogs. More complex relationships were observed for RDW, WBC, lymphocytes, monocytes and platelet counts, with variable relative values below, at and above those of matched controls, depending on age. Significant relative differences (*p* < 0.002) were also observed between dogs with DCM and healthy controls for the clinical biochemistry parameters total protein, globulin, ALT, ALP, blood urea nitrogen (BUN), creatinine and inorganic P. Compared to matched controls, ALT, ALP, BUN and inorganic P were often increased. More complex relationships were observed for total protein, globulin and creatinine, with variable relative values below, at and above those of matched controls, depending on dogs’ age.

## Discussion

The data from the feeding study indicate that feeding healthy adult Labrador retriever dogs a diet containing a combination of the main ingredients kangaroo, peas, red and green lentils – with a total legume inclusion of 60% – sunflower oil, and flaxseed meal for a period of 30 days caused a rapid reduction in RBC, hyperphosphatemia, and possible implications on taurine status, as indicated by reduced urinary concentrations of taurine. The clinical relevance of the hematology and clinical biochemistry findings from the feeding study was at least partially substantiated by interrogation of EHRs of dogs at Banfield® Hospitals with DCM in their diagnosis: significantly decreased RBC counts, HCT and total HGB, and increased plasma inorganic phosphate were observed compared to age and breed-matched healthy controls. While compelling, limitations with the EHR data were a lack of diet history, limited information on how DCM was diagnosed, and potential inaccuracies and confounding factors that may influence measured analyte values. When building the master dataset, only the current patient status was considered, while any previous diagnosis or co-morbidities that may influence measured analytes were not assessed. Attempts to apply such additional filters would require considerable resource in interrogating written records, substantially reduce the dataset, and detrimentally impact the model accuracy, and were therefore not undertaken when the survey was conducted. Also, additional exclusion for comorbidities is often not possible when applying the model in a practice or screening application. For some scenarios it is therefore our preference to define cases and controls as described.

**Fig. 3:**
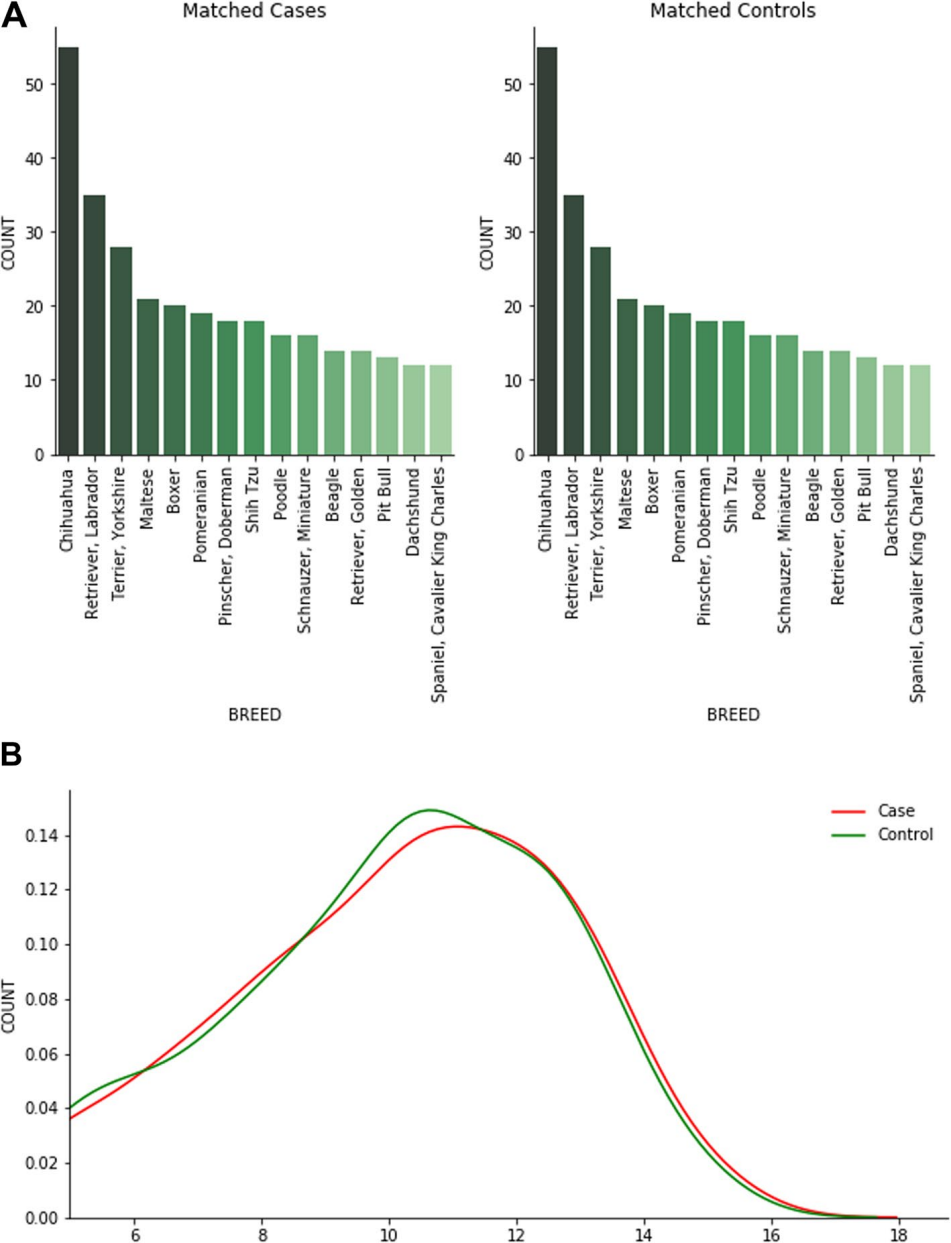
Distribution of cases and controls in the electronic health record survey of Banfield® Pet Hospital patients. **A** The 15 most common breeds for the case and control subsets. The case-control dataset was balanced by breed, with gender and age used as further matching features. **B** A kernel density estimate (KDE) showing the distribution of ages for cases and controls within the case-control dataset

Given the assumption that the Banfield® dogs diagnosed with DCM reflect all causes with differing mechanisms leading to DCM, it is compelling that the blood analytes from the EHR of DCM cases largely mirrored those of the dogs fed the Test diet in the feeding study. Breeds genetically predisposed to DCM were relatively under-represented among the those included (Fig. 3A), which may suggest that many of the DCM cases were diet-related during the time that the survey was conducted. On the other hand, the mixed etiologies were a likely source of dilution of the data and may explain inconsistencies within and between the datasets.

A similar rapid decrease in RBC and increase in plasma inorganic P as observed from day 3 of the feeding study was also recently observed in beagles following 7 days of feeding a high protein diet containing split peas (9th listed ingredient) and lentils (11th listed ingredient) by Reis et al. [22]: mean RBC of ~ 6*10^12^/L and serum inorganic P of 1.67 mmol/L were observed. Specific inclusion levels of the legumes were not provided. However, the fava bean-containing diets at 30% inclusion level tested in the study also decreased RBC similarly compared to the same diets containing fermented fava beans, while plasma P was largely unaffected [22]. On the other hand, no changes in RBC and only a transient increase in plasma inorganic P at 13 weeks were observed in Labrador Retrievers in the 6-month Donadelli et al. study [21]. This may be due to different legume levels in the test diet in that study, which were not specified, although whole lentils and peas were the third and sixth listed ingredient, respectively. Also, the first sampling time in the Donadelli study [21] was at 13 weeks following transition to the test diet, and any earlier, transient changes were therefore not detected.

Iron deficiency was considered as a potential cause of the rapid decrease in HGB and RBC in the Test group. However, the iron concentration was higher in the Test compared to Control diet. The iron sources were from raw materials only in the Control and a combination of raw materials and a premix containing an iron amino acid chelate in the Test diet. Thus iron bioavailability was considered high for both diets. Hemolysis as a cause cannot be completely ruled out, although it was not registered as an issue during plasma analysis or signs observed (see also below) in the EHR survey’s total bilirubin (TBILI) data. However, this area deserves further investigation as released heme can cause oxidative damage by radical oxygen species, impaired nitric oxide signalling, endothelial dysfunction and systemic vasoconstriction, which may impact heart structure and function [27]. Feeding grain-free diets and diets containing peas, lentils or potatoes to dogs has recently been linked to increased median cardiac troponin I and plasma taurine, both suggested by Adin et al. to originate from damaged cardiomyocytes, while no changes in whole-blood taurine or NTproBNP were observed [18].

Although anemia has not previously been suggested as a cause of DCM in dogs, reduced RBC numbers can reduce oxygen transport, which may put undue strain on the heart muscle when increased blood volumes are required to meet cardiac and peripheral tissue needs for oxygen. Anemia may also exacerbate signs and complicate recovery in DCM patients if not discovered and treated. On the other hand, the data from the feeding study and EHRs do not indicate clinically apparent anemia, as reductions in both RBC and HCT below reference ranges (RBC < 6*10^12^/L and HCT < 40) were observed in only a small proportion of test animals (Table 2, Fig. 2).

High choline supplementation of 8-10 mg choline chloride/kg BW have been reported to cause reductions in RBC in dogs [28, 29]. However, the basal diet without supplementation was not tested in those studies and other dietary factors can therefore not be ruled out as a cause of the reduced RBC. On the other hand, McKibben et al. [30, 31] supplemented puppy diets with 1500 and 2000 mg choline chloride/kg diet, respectively, without observing reductions in RBC. In the current study, the average choline intake was 24 mg/kg BW for the dogs fed the Control diet and 44 mg/kg BW for those fed the Test diet, both higher than the supplementation concentrations provided for the dogs in the studies by Davis [28, 29], but far lower than in the McKibben et al. studies [30, 31].

Anti-nutritional factors (ANFs) present in the legumes of the Test diet cannot be ruled out as causes of the reduced RBC. Although not analysed in the diets, phytic acid can bind minerals such as iron and thereby reduce bioavailability. The amphipathic nature of saponins that are present in some legumes are known to cause hemolysis by disrupting cell membranes and changes in intestinal permeability in various animals tested [32–36]. Likewise, legume lectins can agglutinate cells, including red blood cells, by binding to carbohydrate moieties on the cell surface, and also affect intestinal permeability [37]. However, reduced RBC or other hematological changes due to flaxseed inclusion in the form of linseed cake and its inherent cyanogenic glycosides linostatin and neolinostatin in canine diets have not previously been described [38]. The anti-nutritional factors and/or fibre present in the legume-rich Test diet may also impact taurine and other nutrient bioavailabilities, and thus impact cardiac health. If one or more deficiencies in nutrients specifically affecting heart function and health take longer to manifest, this may explain the development of DCM in dogs when fed such diets over longer periods of time.

The increasing number of individuals affected and increasing severity of hyperphosphatemia observed in the Test diet-fed dogs from day 3 of the feeding trial may be a sign of hemolysis, with the disruption of red blood cells causing intracellular phosphate to leak into the plasma. However, and as mentioned above, no direct signs of hemolysis were observed. Alternatively, the increased plasma inorganic phosphate may also be a sign of increased P availability for absorption. Changes in mineral interactions in the intestine, possibly caused by e.g. high phytic acid and fibre in legume-rich diets, may explain differences in P availability. This because: 1) the high plasma P was most likely not caused by dietary P levels, as the Test diet contained about 1.4 g/1000 kcal (0.49%) compared to 2.5 g/1000 kcal (0.91%) in the Control diet, and 2) transient disruptions in plasma concentrations of Ca and iCa were also evident. A hormonal response, e.g. parathyroid hormone (PTH), fibroblast growth factor 23 (FGF-23) and/or vitamin D metabolites, elicited by the low plasma Ca/iCa and/or high plasma P, would be expected in an attempt to restore homeostasis, as indicated by more or less restored concentrations of blood Ca at day 28. Unfortunately, hormonal regulation was not investigated in the current trial. Despite the hyperphosphatemia, no signs of renal marker changes were observed in the Test group dogs, with serum creatinine and urea, as well as urine specific gravity (data not shown) within reference ranges. A small increase in ALP was observed in the Test diet-fed dogs, however as bone-specific ALP did not differ between the dog groups, it is unlikely that resorption of P from bone was a cause of the increased plasma inorganic phosphate concentrations. Again, the inorganic phosphate and ALP data differed in the Donadelli et al. study [21], in which the transient increase in inorganic phosphate at 13 weeks did not exceed the upper reference range and decreases in ALP were observed in dogs fed the legume-rich Test diet following 13 and 26 weeks of feeding.

Although hyperphosphatemia has not been previously cited as a cause of DCM in dogs, it has been associated with myocardial hypertrophy, bone restructuring, and possibly reduced hematocrit in parathyroid hormone-treated, parathyroidectomized and 5/6 nephrectomized Wistar rats fed high-P (1.2%, Ca:P 0.58) diets [39]. Because the experimental conditions differ substantially, including species investigated and dietary P and Ca:P levels, it is difficult to directly compare our results with those reported in Neves et al. [39]. In other studies, high FGF-23 concentrations in human patients suffering from chronic kidney disease has been implicated in the pathogenesis of left-ventricular hypertrophy [40]. FGF-23 was subsequently shown to cause cardiac myocyte hypertrophy in vitro [41], which has later been demonstrated in mice in vivo [42]. Again, how this relates to the current study is difficult to assess at this time, but closer inspection of hormones regulating mineral homeostasis should be included in future trials with dogs fed alternative diets or in clinical trials with dogs with clinically apparent DCM.

Taurine concentrations in whole blood and plasma were not significantly reduced in the Test group dogs, confirming recent observations in dogs suffering from apparent diet-associated DCM [2] as well as healthy dogs fed grain-free diets [16, 21]. Concentrations were transiently increased on day 14 in the current study, possibly due to up-regulated biosynthesis from cysteine. However, decreased urinary taurine concentrations, a trend not observed previously [16, 21], indicated that neither biosynthesis nor taurine potentially released from cardiomyocytes [18] could fully meet taurine requirements when fed the Test diet. This may have been compounded by dietary methionine and cysteine levels only marginally meeting recommended allowances in this diet. An increased need for cholesterol and taurine to meet increased bile salt synthesis as a response to increased fecal bile acid/salt losses, as previously reported [21], may therefore have contributed to the decreases in plasma cholesterol and urinary taurine. Interestingly, the whole blood taurine concentrations at baseline and day 3 for both diet groups and day 28 for the Test diet group were below, while plasma concentrations were within, the reference ranges recently suggested for Golden retrievers (213-377 and 63-194 μmol/L, respectively) [43]. Although these reference ranges established for Golden retrievers need to be validated for other dog breeds, the data provided herein suggest they may be of value for Labrador retrievers. As previously suggested [21, 23], whole blood and urinary taurine excretion, as assessed by taurine:creatinine ratio, appear to be more sensitive measures of taurine status in dogs than plasma taurine, and relevant for evaluating taurine status in future feeding studies.

This study had limitations, including the small number of dogs included in the feeding study; the limited data regarding hemolysis; the absence of reticulocyte count and cardiac troponin I data; and the above-mentioned limitations in the EHR data with the lack of diet history, limited information on how DCM was diagnosed, and factors that may have influenced analyte values.

## Conclusions

The data from the 30-day, longitudinal feeding study, indicate that the Test diet containing kangaroo, 60% of the legumes peas (20%) and lentils (40%), and flaxseed (7%) may cause reduced RBC and hyperphosphatemia, as well as possible disturbances in taurine status. EHR interrogation of dogs with DCM in their diagnosis largely corroborated the hematology and clinical biochemistry findings from the feeding study, suggesting these data may be clinically relevant. Further studies are needed to increase our understanding of the relevance of the findings and further details regarding the pathophysiology associated with the development of diet-associated DCM in dogs.

## Materials and methods

### Feeding study

#### Study design

The study (project nr. 69780) was overseen and approved by the Institutional Animal Welfare and Ethical Review Body and conducted under the authority of the UK Animals (Scientific Procedures) Act 1986.

The eleven dogs included in the study were selected from the colony at Waltham Petcare Science Institute following behavioural assessments and health-screening, including assessment of blood hematology, biochemistry and urinary health parameters, as well as a cardiac ultrasound scan conducted on conscious dogs by a board-certified cardiologist. Only dogs with health parameters within normal ranges and without signs of cardiac disease were considered for inclusion. One male dog was excluded from the study due to signs of cardiac changes observed during screening. A replacement could not be identified, and the Control groups was therefore limited to five dogs. All study dogs were neutered adult Labrador retrievers, three males and eight females, from a total of seven litters, aged between 2.5 to 7.7 years (mean 5.8 years; median 5.5 years). In a longitudinal study design, the dogs were randomized into one of two diet groups – Control or Test – balanced for sex, age, body weight (BW), familial affiliation (litter mates) and energy requirements. The Control group comprised five and the Test group six dogs with mean body weights of 25.4 (range 22.4-28.8) and 25.3 (range 21.5-33.9) kg and mean energy intakes of 1311 (range 925-1947) and 1105 (range 875-1698) kcal per day, respectively, at the start of the study. Due to time and resource constraints, a pre-feed period of 2.5 week period was implemented in which all dogs were fed the Control diet for the purpose of gaining baseline measurements. Subsequently, the dog groups were transitioned to the respective dry format experimental diets over a period of 5 days, and thereafter fed exclusively the respective diets for 30 days.

#### Housing and feeding

All dogs were housed according to diet group to minimize risk of feeding error and potential impact of coprophagia. The amount of diet provided was according to each dog’s estimated metabolizable energy requirements (MER) needed to maintain ideal BW and body condition score (BCS) according to the 9-point scale [44]. Dogs received one morning meal supplying 60% and an afternoon meal supplying 30% of MER with the remaining diet allowance of 10% as training rewards. Water was freely available to dogs at all times. Within their diet groups, the study dogs were exercised and socialized as normal and according to institutional practices.

#### Diets

Single batches of two extruded dry format diets were specifically formulated and manufactured for this study (Mars Petcare, Franklin, Tennessee, USA). The Control diet contained ingredients commonly used in conventional diets and did not contain pea, lentil or flaxseed products: ground wholegrain corn, poultry by-product meal, corn gluten meal, animal fat, meat and bone meal, soybean meal, ground wholegrain wheat, brewers rice, chicken by-product meal, dried beet pulp, methionine, vitamins and minerals. The Test diet, which was specifically formulated with less conventional ingredients compared to the Control diet, contained kangaroo, split peas, red lentils, green lentils, sunflower oil, flaxseed, pea fibre, methionine, vitamins and minerals. The inclusion level of the legumes split peas, red lentils, and green lentils was 20% each, with a total of 60%, on an as fed basis. Both were formulated for compliance with AAFCO [24] and NRC [25] recommended allowance for adult dogs. The nutrient composition analysis of both diets (Table 1; as-fed basis) was carried out in parallel by Eurofins (USA) and according to the Association of American Feed Control Officials guidelines [24].

Cyanogenic glycoside (linamarin, linustatin, neolinustatin) content of the diets were determined by HPLC-UV (UpScience, France).

#### Sampling and analyses

At baseline and days 3, 14 and 28 following transition onto the trial diets, fasted blood (8.5 mL; at least 12 h since last meal) was drawn from the jugular vein. Samples were kept on ice until processing and/or analysis was performed (within 60 min of sampling). Free-catch (at baseline and days 3, 14 and 28) and pooled 4-d urine collections before baseline and days 27-30 were also sampled. Any samples that could not be analysed immediately were stored at − 80 °C for a maximum of 2 years.

The differing days for blood, free-catch urine and pooled urine sample collections at the end of the trial were necessary for logistical reasons, and therefore reference to samples from both day 28 and 30 are made to reflect this. Heart ultrasound scans were performed to screen all dogs for trial inclusion, while due to unforeseen circumstances (cardiologist’s illness on the scheduled second visit), scans were only performed on six of the 11 dogs at the end of trial.

Standard hematology was measured in EDTA-dosed blood samples with a Mythic cell counter (Woodley Veterinary Diagnostics, Horwich, UK). Plasma was separated from lithium heparinized blood by centrifugation (1999 g for 10 min at 4 °C) and samples were analysed for standard biochemistry (Olympus AU480 Biochemistry analyser; Olympus Europe GmbH). The instruments were maintained according to the manufacturer’s schedule. Performance of each machine was verified by analysis of manufacturer’s QCs prior to and post-sampling. In addition, the AU480 is further verified by inclusion on an external quality assurance program (RIQAS, Randox).

Ionized Ca was measured in lithium heparinized whole blood within 30 min of collection by an ion-selective electrode of a blood gas-analyzer (Stat Profile Prime® Critical Care Analyser, Woodley Equipment Company LTD, Horwich, UK). Performance of the instrument was verified by analysis of the manufacturers’ tri-level quality control material before and after sample measurement.

Bone-specific ALP was analysed using the BAP MicroVue™ Quidel enzyme linked immunosorbent assay (ELISA; TECO Medical Group) according to the manufacturer’s instructions. Frozen (stored at − 80 °C) samples were sent to IDEXX Bioanalytics (Ludwigsburg, Germany) for NTproBNP analysis (ELISA).

For taurine analysis, plasma stored at − 80 °C was defrosted before analysis, frozen whole blood samples underwent two freeze thaw cycles and were diluted with deionized water to promote cell lysis, while fresh and pooled urine samples were diluted 1:5 with sample diluent buffer (5% SSA). For all sample types, sample processing buffer (5% SSA+ 500μMol/L Norleucine+ DGAA) was added 1:1, mixed using a Vortex Whirlimixer and allowed to incubate at room temperature (RT) for 20 min. Sample was then centrifuged at 7000 *g*, for 5 min at RT. Supernatant was removed and filtered with a Whatman Mini-Uniprep Syringeless 0.2 Filter. Analysis was carried out in duplicate on the same day as sample processing on a Biochrom 30+ Amino Acid Analyzer (Biochrom Ltd).

### EHR interrogation

Banfield® Pet Hospitals are one of the largest networks of primary care veterinary hospitals within the United States with over 1000 practices across 42 states. Banfield® maintains a centralized electronic medical record system that stores information from the millions of patient visits since the mid-1990’s. Data were extracted from the anonymised EHRs of client-owned dogs visiting Banfield® Pet Hospitals between January 2, 2018, and December 31, 2019. The dataset was fully anonymised by removing any client and animal details that might be personally identifiable. Records were filtered to only include pets between age 4 and 16 years (inclusive) and with both veterinary visit and laboratory test data (hematology and biochemistry), this pairing was defined as laboratory results 90 days before or after a visit. This filtered data set contained 39,574 medical records for 32,127 unique dogs.

This data extract contained Banfield® diagnosis codes, breed, weight, gender, neuter status and age. The laboratory test data included for hematology were RBC, HCT, HGB, MCV, MCH, MCHC, RDW, WBC, lymphocyte %, monocyte %, eosinophil %, PLT, and MPV; and for clinical biochemistry total protein, albumin, globulin, ALT, ALP, creatinine, blood urea nitrogen (BUN), cholesterol, glucose, Ca, inorganic P, and TBILI. Drift and variability in certain analytes (RBC, HCT, HGB, MCV, MCH, MCHC and RDW) can be observed in hospital analysers. To mitigate this variability and minimize drift in analysers used, hospitals are instructed to use a regular programme of deep cleaning, calibration, and ongoing monitoring. However, caution should always be exercised when interpreting results involving those analytes.

Prior to case-control matching, imputation was used to infill missing data from records that contained at least one missing blood analyte value, this accounted for 0.47% of records. To do this, the IterativeImputer function from SKlearn package v0.24.2 was applied, which uses a BayesianRidge regressor. To ensure that imputed values are biologically valid, a minimum imputation threshold of 0 has been provided as a parameter. This threshold ensures that no imputed values below 0 can be returned by the algorithm.

Pets were identified as cases using the diagnoses terms “Canine Dilated Cardiomyopathy” or “Congestive Dilated Cardiomyopathy” in the Banfield® database. Records of patients with DCM were matched with healthy control pets without DCM (attending for a routine wellness visits) using propensity score matching. The Pymatch v0.3.4 (GitHub - benmiroglio/pymatch) Python library was used to create matched cases and controls using breed, gender (including neutered status; data not shown) and age (rounded to the nearest integer year). Subsequently, the case-control dataset consisted of 840 records, 420 for cases and 420 for controls. The two distributions showing the most common breeds are depicted in Fig. 3A. In Fig. 3B, a kernel density estimate (KDE) shows the distribution of ages.

### Statistical analysis

#### Feeding study

Comparisons within all measures generated were tested at an overall significance level of 5%. Linear mixed effects models were fit to the data for all measures. Fixed effects were diet group, time point (day) and their interaction, and animal was the random effect. Log_10_ transformation of the response was applied if it appeared necessary from visual inspection of the residual distribution. Means with 95% confidence intervals are reported for all combinations of diet group and day (and post prandial time point where appropriate). Within each diet group, comparisons between all post baseline time points and baseline are reported with confidence intervals and *p*-values. The differences over time were then compared between diet groups and are also reported with confidence intervals and *p*-values. Comparisons for measures that were log_10_ transformed are reported as fold changes, whereas those without a transformation are reported as differences on the measured scale. All analyses were performed using R version 3.4.3 (2017-11-30).

#### EHR interrogation

Statistical differences between pets with DCM (cases) and healthy pets (controls) within the laboratory test data were identified by applying generalized additive models (GAMs) and generalized additive mixed models (GAMMs), a branch of linear modelling that can capture nonlinear patterns within data.

The step-wise process used to model each analyte, where smoothing has been achieved using thin plate spline smoothing, was performed as follows. Data were extracted for the required analyte and erroneous zero values excluded (orphaned paired data removed also). A Basic GAM model was generated using R3.6.1 [45] and the R package mgcv [46] to predict the value of each analyte using smoothed age, without random effects or the DCM term. This step was applied to investigate whether the residuals improved if the variable was log transformed (using natural log) and to also identify outlying observations (residual > 4, which were subsequently removed). A plot of the data for each analyte at this stage, together with loess smoothed trend lines for each value of the DCM flag, can be found in Fig. 2. This was plotted using the R package ggplot2 [47].

A GAMM model was fit, again using mgcv, with DCM as main effect and a smooth interaction between age and DCM Flag, and random term for the matched pair (main model). Additionally, for each analyte a secondary model was also fit, the same as main model but with Sex and an Age*Sex interaction, and the improvement of fit compared to the main model assessed. As no analyte showed a significant improvement in fit, no Sex effects were included in the final models.

For each analyte, the most significant *p*-value out of the individual *p*-values for the DCM Flag main effect and the *p*-values for the smoothing terms involving the DCM flag (from the main model) were taken as a conservative bound on the overall DCM *p*-value. Graphs were also produced for each analyte, again using ggplot2, showing the estimated difference between a positive and negative DCM flag by age, shown in Fig. 1. To evaluate whether a variable was statistically significant, a Bonferroni correction was used when interpreting *p*-values and constructing confidence intervals. Therefore, *p*-values < 0.05/25 = 0.002 were considered statistically significant, where 25 represents the number of blood analytes.

## Data Availability

The datasets used and/or analysed during the current study are available from the corresponding author on reasonable request.

## Abbreviations

ANF: Anti-nutritional factors
BUN: Blood urea nitrogen
BW: Body weight
CI: Confidence interval
DCM: Dilated cardiomyopathy
EHR: Electronic health record
FGF23: Fibroblast growth factor 23
iCa: Ionized calcium
MER: Maintenance energy requirement
NTproBNP: N-terminal pro b-type natriuretic peptide
P: Phosphorus
inorganic P: Inorganic phosphate
