# Correction: Responses in randomised groups of healthy, adult Labrador retrievers fed grain-free diets with high legume inclusion for 30 days display commonalities with dogs with suspected dilated cardiomyopathy

**DOI:** 10.1186/s12917-022-03291-8

**Published:** 2022-05-17

**Authors:** Anne Marie Bakke, Joshua Wood, Carina Salt, David Allaway, Matt Gilham, Gail Kuhlman, Tiffany Bierer, Richard Butterwick, Ciaran O’Flynn

**Affiliations:** 1 Waltham Petcare Science Institute, Mars Petcare UK, Freeby Lane, Waltham-on-the-Wolds, Melton Mowbray, Leicestershire, LE14 4RT UK; 2 Mars Petcare US, Brentwood, TN USA

**Correction: BMC Vet Res 18, 157 (2022)**

**https://doi.org/10.1186/s12917-022-03264-x**

Following the publication of the original article [1], the authors are requesting to update the Competing Interests section. The previous statement “The authors declare that they have no competing interests” needs to be updated to “The authors declare that they have no competing interests. All authors were employees of Mars Petcare at the time that the studies were conducted; Anne Marie Bakke, Joshua Wood, Carina Salt, David Allaway, Matt Gilham, Richard Butterwick and Ciaran O’Flynn at the WALTHAM Petcare Science Institute, and Gail Kuhlman and Tiffany Bierer at Mars Pet Nutrition North America.”.

The updated Competing Interest section is provided below.

**Competing interests**

The authors declare that they have no competing interests. All authors were employees of Mars Petcare at the time that the studies were conducted; Anne Marie Bakke, Joshua Wood, Carina Salt, David Allaway, Matt Gilham, Richard Butterwick and Ciaran O’Flynn at the WALTHAM Petcare Science Institute, and Gail Kuhlman and Tiffany Bierer at Mars Pet Nutrition North America.
